# Beyond Osteoarthritis: A Case of a Massive Intraarticular Ganglion Cyst in the Knee

**DOI:** 10.7759/cureus.79967

**Published:** 2025-03-03

**Authors:** Felix Rivera Troia, Carlos J Perez Lopez, Carlos Perez Cardona

**Affiliations:** 1 Orthopaedic Surgery, Ponce Health Sciences University, Ponce, PRI; 2 Orthopaedic Surgery, Mayaguez Orthopaedics, Mayaguez, PRI

**Keywords:** intra articular ganglion cyst, knee joint, massive ganglion cyst, osteoarthritis, surgery

## Abstract

Intraarticular ganglion cysts of the knee are rare, typically incidental findings, and often asymptomatic. However, large cysts may be of clinical interest due to their potential mass effect on surrounding structures. We present the case of a 54-year-old female with a three-year history of osteoarthritis-related knee pain unresponsive to conservative management who ultimately underwent a total knee arthroplasty (TKA). Preoperative imaging revealed a massive, multiloculated intraarticular ganglion cyst extending from Hoffa’s fat pad to the medial joint capsule. While the cyst did not interfere with the TKA, its size and location raise important considerations regarding the possible role of intraarticular ganglion cysts in osteoarthritis progression and symptomatology. This case highlights the significance of recognizing and characterizing these lesions and reviewing their potential implications in the setting of advanced knee degeneration.

## Introduction

Intraarticular ganglion cysts of the knee, first described by Caan in 1942, are rare occurrences, most commonly developed in the extensor compartment of the distal forearm [1]. However, when found intraarticularly, they have been reported to originate from the cruciate ligaments and menisci, among other locations [2,3]. These mucinous, fluid-filled lesions consist of mucopolysaccharides and are believed to develop due to a combination of repetitive trauma and chronic connective tissue degeneration [4,5]. Some studies suggest that joint degeneration, such as that seen in osteoarthritis, may contribute to the formation of these cysts [6]. These lesions are often incidental findings on imaging or arthroscopy; however, they can occasionally become symptomatic, causing pain, mechanical symptoms, or a restricted range of motion.

Although intraarticular ganglion cysts are uncommon, large multiloculated lesions are even rarer, particularly in patients with advanced osteoarthritis. The clinical significance of such cysts remains unclear, as their symptoms may overlap with underlying degenerative joint disease. However, extensive intraarticular cystic degeneration could be a marker of disease severity or a contributor to joint dysfunction. This case report describes a massive intraarticular ganglion cyst in an osteoarthritic knee, highlighting the importance of recognizing and characterizing these lesions in clinical practice.

## Case presentation

We present a case of a 54-year-old female with a past medical history of hypertension, Sjögren’s syndrome, fibromyalgia, lupus, and rheumatoid arthritis who arrived at the clinic with a 3-year history of left knee pain. Initially, the pain was intermittent, worsening with activity, but had progressively become constant, even at rest. She denied any history of trauma. The patient had been followed in the clinic for several years and had undergone extensive conservative management for osteoarthritis of the knee, including weight reduction, medication, knee strengthening exercises, and two corticosteroid injections, but her symptoms failed to improve.

**Figure 1:**
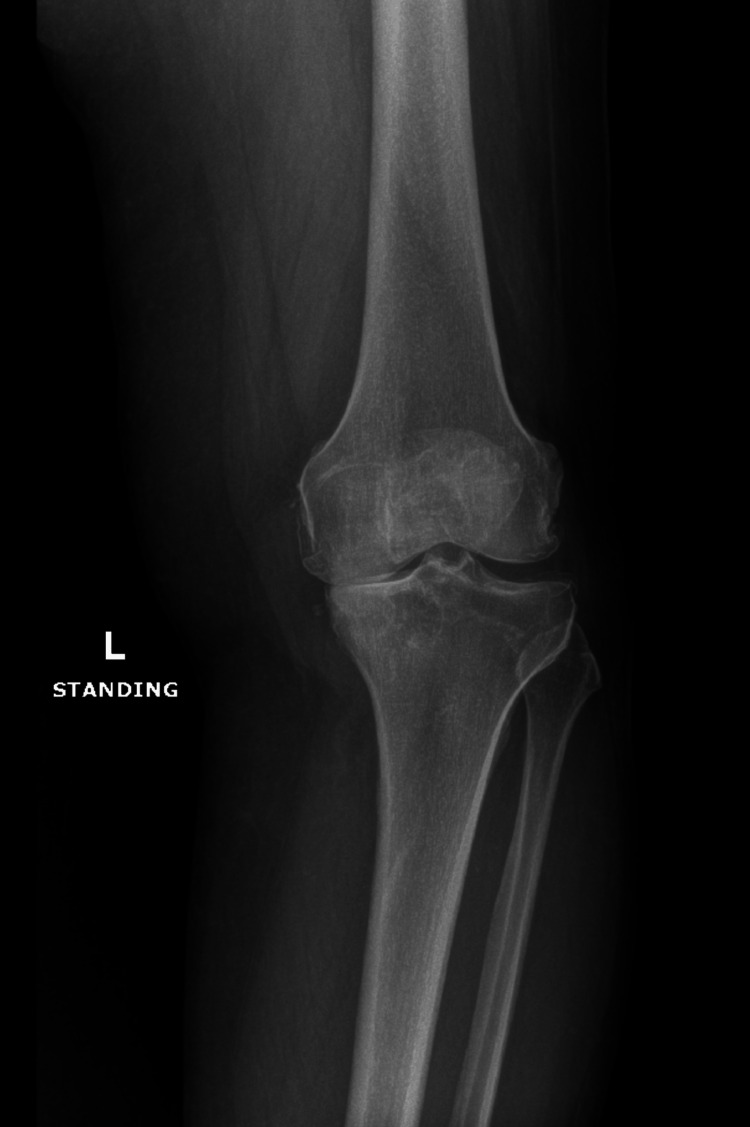
X-ray showing anterior posterior view of the patient's left knee.

**Figure 2:**
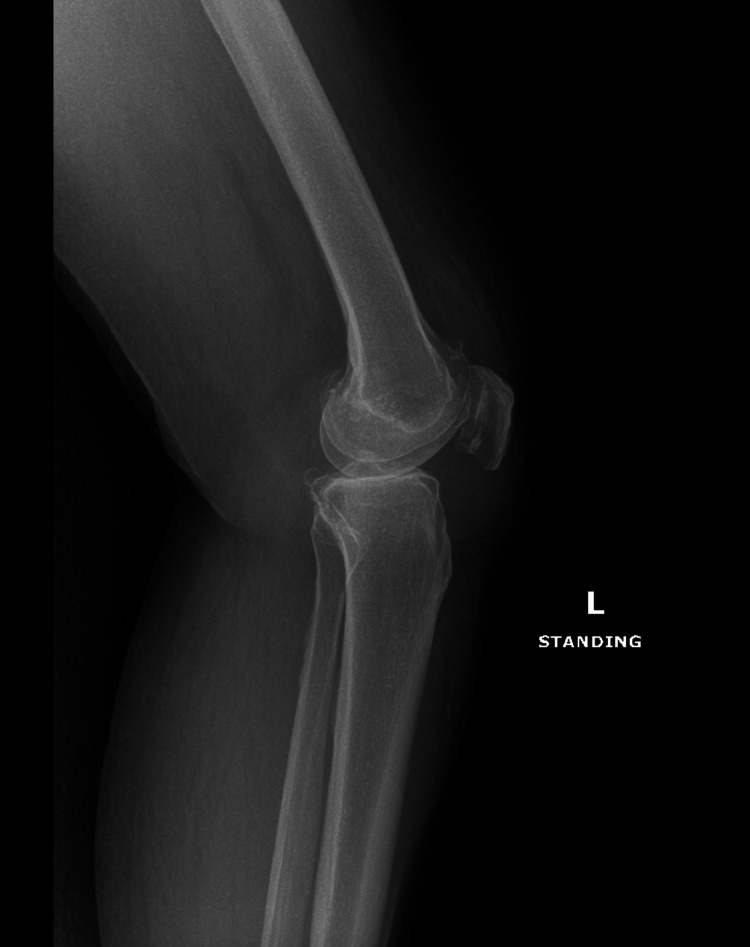
X-ray demonstrating a lateral view of the patients left knee.

On physical examination, the patient’s left knee appeared markedly swollen, accompanied by crepitus throughout the range of motion. Tenderness was noted along both the medial and lateral joint lines. There was significant restriction in the range of motion, and the patient demonstrated a pronounced limp. A notable varus deformity was also observed. Preoperative imaging included standing anteroposterior and lateral radiographs, as well as an MRI of the left knee. Radiographs revealed severe osteoarthritic changes, characterized by marked narrowing of the medial tibiofemoral compartment and the presence of marginal osteophytes, findings that correlated with the patient’s clinical symptoms (Figures 1, 2).

**Figure 3:**
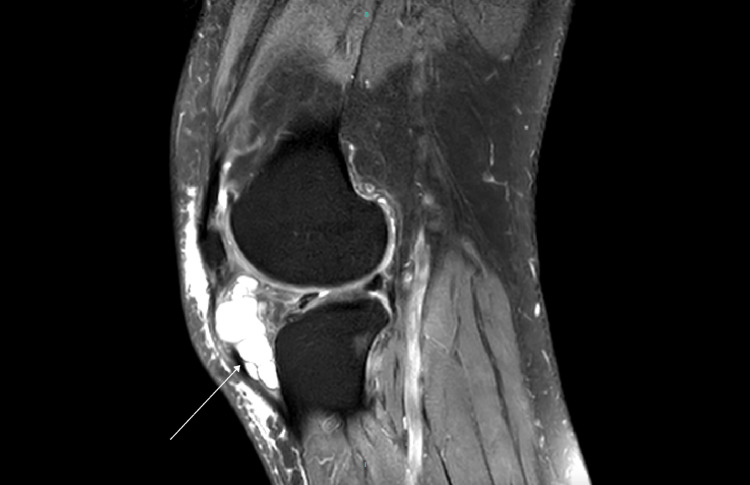
MRI demonstrating sagital view of the left knee with massive cyst extending from Hoffa's fat pad (white arrow).

**Figure 4:**
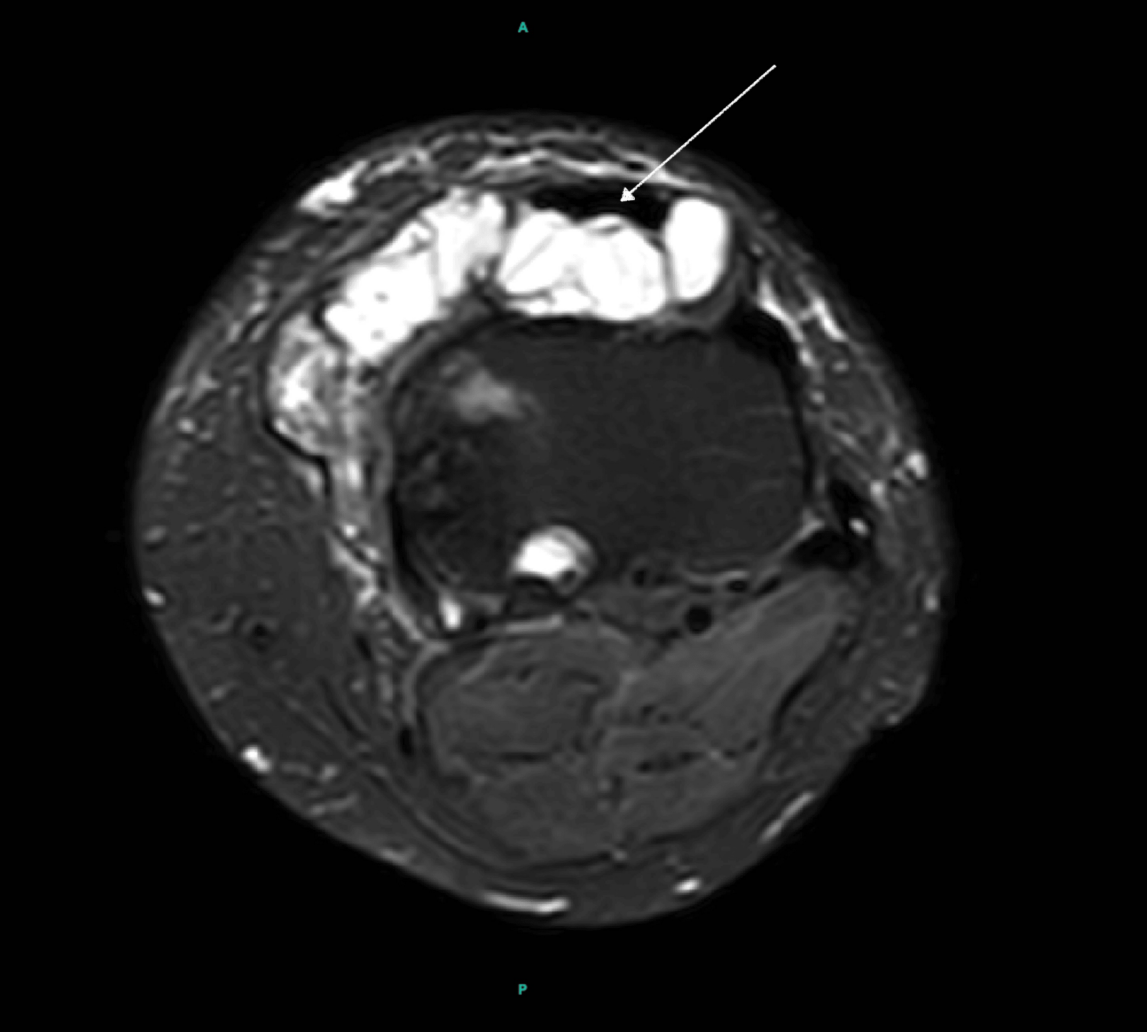
MRI demonstrating axial view of the left knee showing cyst extension into the medial joint capsule (white arrow).

**Figure 5:**
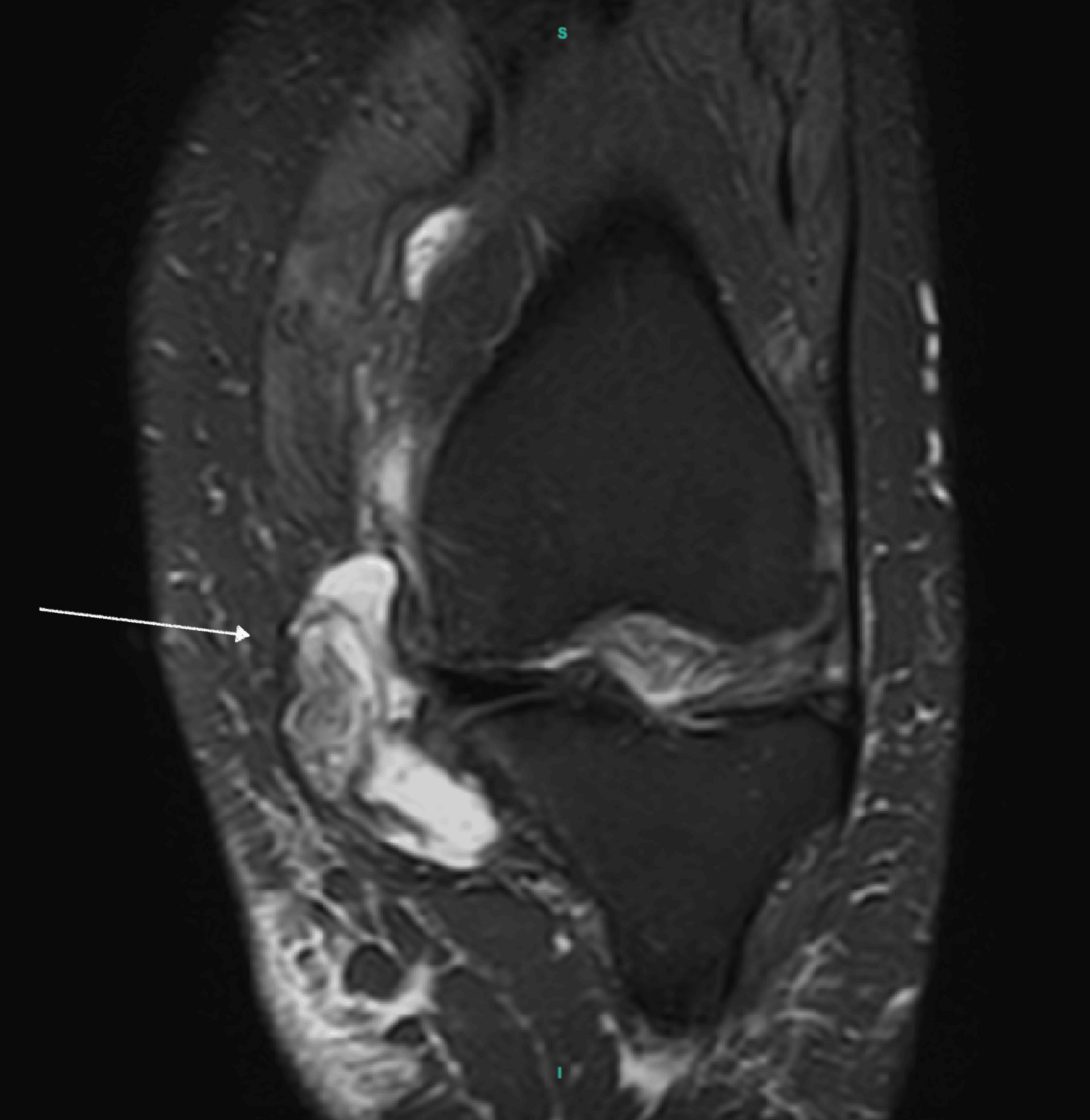
MRI demonstrating coronal view of the left knee showing the cyst having a mass effect on medial joint capsule (white arrow).

MRI demonstrated tricompartmental degenerative changes with extensive cartilage loss in the medial compartment, including grade 4 chondromalacia. Additionally, a large, multiloculated cystic lesion was identified, extending from Hoffa’s fat pad anteriorly and medially to the joint capsule (Figures 3, 4, 5). 

The patient subsequently underwent robotic-assisted total knee arthroplasty with a press-fit implant. The procedure was completed without complications. Additionally, the lesion did not interfere with the surgical technique, implant positioning, or soft tissue balancing. Postoperatively, the patient had an uneventful recovery and progressed well with rehabilitation. While the cyst was an incidental finding, its size and location highlight the importance of recognizing atypical intraarticular pathology in the setting of advanced osteoarthritis.

## Discussion

Intraarticular ganglion cysts of the knee are uncommon findings, often discovered incidentally during imaging or arthroscopy. While their clinical significance still remains a topic of debate, their presence in osteoarthritic knees raises important questions about their role in joint degeneration and symptomatology. Some studies suggest that chronic mechanical stress and synovial irritation, both hallmarks of osteoarthritis, may contribute to the formation of these cysts [4,5]. However, it remains unclear whether they are merely byproducts of the degenerative process or active contributors to joint dysfunction. A reported case of a ganglion cyst within the anterior cruciate ligament suggested a potential association with degenerative processes, further supporting the need for continued investigation into their pathophysiological relevance [7].

Although ganglion cysts are typically asymptomatic, their size and location can influence the types of symptoms experienced. Large cysts within the knee joint have been known to cause mechanical symptoms, such as locking, clicking, and joint line tenderness. The cyst's location can also affect symptom presentation; for instance, anterior cysts tend to cause mechanical disturbances, while posterior cysts are more likely to result in a reduced range of motion [8]. A similar case described a patient with an intraarticular ganglion cyst located deep to the iliotibial band, who presented with knee swelling, joint line tenderness, and reduced range of motion [9]. The patient’s age and clinical presentation closely align with the physical exam findings observed in our case, further emphasizing the importance of considering these lesions as potential contributors to mechanical symptoms and functional limitations in patients with knee pathologies, especially when other common conditions, such as osteoarthritis, may also be present.

The clinical presentation of knee osteoarthritis and intraarticular ganglion cysts can overlap significantly, making it challenging to determine whether symptoms are solely due to the degenerative disease or if the cyst contributes to worsening pain and dysfunction. Osteoarthritis itself is often associated with joint pain, swelling, and mechanical symptoms [10], which are similar to those caused by large ganglion cysts. This overlap can lead to diagnostic uncertainty, as it is not always immediately clear whether the cyst is a primary cause of the patient's symptoms or if it is exacerbating the underlying degenerative process. In cases like this, it becomes important to consider both the patient's clinical presentation and imaging findings to determine the potential role of the cyst in worsening symptoms, particularly when the two conditions coexist. In this case, a large intraarticular ganglion cyst was identified on preoperative imaging in a patient with advanced knee osteoarthritis. Despite its considerable size and multiloculated structure, the lesion did not interfere with the TKA or impact surgical decision-making. This raises an important question: to what extent do these lesions influence pain, stiffness, and overall joint function in osteoarthritis?

## Conclusions

Intraarticular ganglion cysts of the knee are rare, but clinically significant lesions may coexist with advanced osteoarthritis. In this case, we identified a large, multiloculated ganglion cyst in a patient with severe knee osteoarthritis, which was an incidental finding on preoperative imaging. While the cyst did not directly interfere with the total knee arthroplasty, its size and location raise important questions regarding its potential role in symptom exacerbation and joint dysfunction. Although ganglion cysts are typically asymptomatic, their presence in the setting of severe degenerative disease can complicate the diagnostic process, as the symptoms may overlap with those of osteoarthritis. This case highlights the importance of recognizing and characterizing these lesions in patients with knee pathology, as they may contribute to mechanical symptoms and functional limitations. Further research is needed to determine the exact relationship between ganglion cysts and osteoarthritis progression, as well as the clinical implications of their presence in such cases.
